# Clinical Prediction Rule for Stratifying Risk of Pulmonary Multidrug-Resistant Tuberculosis

**DOI:** 10.1371/journal.pone.0012082

**Published:** 2010-08-11

**Authors:** Dalila Martínez, Gustavo Heudebert, Carlos Seas, German Henostroza, Martin Rodriguez, Carlos Zamudio, Robert M. Centor, Cesar Herrera, Eduardo Gotuzzo, Carlos Estrada

**Affiliations:** 1 Institute of Tropical Medicine “Alexander von Humboldt”, Universidad Peruana Cayetano Heredia, Lima, Perú; 2 Division of General Internal Medicine, University of Alabama at Birmingham, Birmingham, Alabama, United States of America; 3 Birmingham Veterans Affairs Medical Center, Birmingham, Alabama, United States of America; 4 Peruvian Tuberculosis Program, Ministry of Health, Lima, Perú; 5 Division of Infectious Diseases, University of Alabama at Birmingham, Birmingham, Alabama, United States of America; McGill University, Canada

## Abstract

**Background:**

Multidrug-resistant tuberculosis (MDR-TB), resistance to at least isoniazid and rifampin, is a worldwide problem.

**Objective:**

To develop a clinical prediction rule to stratify risk for MDR-TB among patients with pulmonary tuberculosis.

**Methods:**

Derivation and internal validation of the rule among adult patients prospectively recruited from 37 health centers (Perú), either a) presenting with a positive acid-fast bacillus smear, or b) had failed therapy or had a relapse within the first 12 months.

**Results:**

Among 964 patients, 82 had MDR-TB (prevalence, 8.5%). Variables included were MDR-TB contact within the family, previous tuberculosis, cavitary radiologic pattern, and abnormal lung exam. The area under the receiver-operating curve (AUROC) was 0.76. Selecting a cut-off score of one or greater resulted in a sensitivity of 72.6%, specificity of 62.8%, likelihood ratio (LR) positive of 1.95, and LR negative of 0.44. Similarly, selecting a cut-off score of two or greater resulted in a sensitivity of 60.8%, specificity of 87.5%, LR positive of 4.85, and LR negative of 0.45. Finally, selecting a cut-off score of three or greater resulted in a sensitivity of 45.1%, specificity of 95.3%, LR positive of 9.56, and LR negative of 0.58.

**Conclusion:**

A simple clinical prediction rule at presentation can stratify risk for MDR-TB. If further validated, the rule could be used for management decisions in resource-limited areas.

## Introduction

Multidrug-resistant tuberculosis (MDR-TB), defined as resistance to at least isoniazid and rifampin, is a growing problem [1]. Worldwide, the number of MDR-TB cases reached an estimated 390,000–510,000 in 2008, or 3.6% of all incident TB cases [2]. The global expenditures in diagnosis and treating MDR-TB and extremely drug-resistant tuberculosis (XDR-TB) were estimated at U.S. $700 million for 2009 [3]. The cost of treating a person with MDR-TB is estimated to be 50 to 200 times higher than treating a patient with drug-susceptible TB [2], or $10,000 versus $100 for a susceptible TB case [3].

Culture with drug susceptibility testing (DST) and molecular markers are essential for properly managing drug susceptible and MDR-TB. Unfortunately, such tests are lengthy, costly, and not universally available in resource-limited settings, which bear the major burden of MDR- and XDR-TB. WHO estimates that up to 96% of patients with MDR-TB are not being diagnosed and treated according to international guidelines [4], [5], [6]. Therefore, it is imperative to develop new methodologies for faster and affordable DST, as well as low-cost techniques, for easier identification of patients at risk for MDR-TB.

Clinical prediction rules (CPR) are simple, standardized clinical tools that utilize components of history, physical examination and basic testing to stratify risk, help make a diagnosis, or predict an outcome [7], [8], [9]. In tuberculosis, CPR were developed to focus on infection control decisions [10], [11], [12], the diagnosis of smear-negative pulmonary TB [13], and prognosis [14]. In a recent retrospective study, a CPR was developed to predict the presence of drug-resistant TB in a high HIV prevalent area – Thailand [15]. We developed a clinical prediction rule, with prospectively collected data, to stratify risk for MDR-TB among patients with pulmonary tuberculosis.

## Methods

### Ethics Statement

The Ethics Committee of *Universidad Peruana Cayetano Heredia* approved the clinical trial protocol, where all patients provided written informed consent for their information to be stored in the hospital database and used for research. The Ethics Committee of *Universidad Peruana Cayetano Heredia* and the Institutional Review Board of the University of Alabama at Birmingham approved the use of the existing data for the purposes of this study (patient consent was not obtained again).

### Design and Patients

We derive the clinical prediction rule from data collected in a prospective cohort study, from a phase 3 clinical trial to evaluate rapid diagnostic tests for MDR-TB conducted in Lima, Perú from May 2004 to June 2005 [16]; patients were enrolled from 37 health care centers. The health centers are located in poor shantytown areas of Lima (population 7.5 million). Patients were enrolled in the trial if: a) there was a clinical suspicion of pulmonary TB with an initial positive acid-fast bacillus (AFB) smear, or b) had failed therapy or if they had a relapse within the first 12 months; all patients underwent a sputum culture. At the time the trial was conducted, the prevalence of MDR-TB was 5·3% in the area of Lima where it was executed [17].

The data obtained comprises demographic characteristics, risk factors for acquiring MDR-TB, associated conditions, symptoms, physical exam and radiographic findings. The risk factors studied were history of prior TB (failed therapy or relapse within the first 12 months of standard therapy) [18], [19], [20], [21], [22], MDR-TB contact [20], HIV [18], [20], history of imprisonment [21], [22], [23], and health care workers [20], [24]. Physical exam findings were classified as normal or abnormal; for example, for the lung exam, the presence of crackles, decreased breath sounds, or other abnormalities. Study physicians classified chest radiographic findings and supplemented data collection by concurrent medical record review. Susceptibility testing was performed by the indirect proportion method on Lowenstein-Jensen media at the National Institute of Health in Lima; personnel performing the culture were unaware of these clinical or radiological characteristics.

### Statistical Analysis

The analytical strategy was as follows: first, we defined the patients to be included in the dataset to derive the CPR. Then, we performed bivariate analyses of all of the candidate variables with culture positive MDR-TB as the main outcome of interest. The third step was building a logistic regression model including the variables identified in the bivariate analyses. Finally, we conducted analyses to determine the discrimination characteristics of the tool and tested its validity and robustness.

### Score Derivation

Patients included in the derivation cohort were at least 18 years of age, had an initial suspicion of pulmonary TB with a positive AFB smear, and had culture-proven pulmonary TB.

We performed bivariate analyses using the chi-square test for nominal categories and the t-test for continuous variables to narrow the list of potential predictor variables.

We then performed a forward logistic regression model using candidate variables that had the two following characteristics: first the variable had to be identified in the bivariate analyses at a p level of <0·20; second this variable had to be present in at least 5% of the study sample. Variables with a p level of <0·05 were retained in the final model. Only patients with complete data on all covariates were included in this step.

We calculated the area under the receiver operating curve (AUROC) after computing the predicted probability for each patient using the logistic regression coefficients to assess the discrimination of the model.

The final model was used to derive a simple and clinically applicable risk score. We assigned 1 point to the smallest regression coefficient and serving it as the least common denominator for assigning point values for the score items; then we rounded it up to the next integer as described by Le Gal and colleagues [25]. To avoid negative numbers in the overall score, we added a minimum integer to re-scale the lowest value to zero. We then explored the predictive accuracy of the score by the proportion of patients with pulmonary MDR-TB in each category (prevalence or pretest probability) and calculated the 95% confidence intervals for incremental likelihood ratios [26].

### Model Validation

We performed internal validation of the modeling approach and the final model in two ways. First, we tested the predictive ability of the model by determining the goodness-of-fit with the Hosmer-Lemeshow test; a p-value >0·05 suggests a non-significant discrepancy between the observed and predictive events. Second, we bootstrapped the full model 2,000 times and obtained bias-corrected and accelerated (BCa) 95% confidence intervals to assess the stability of the regression coefficients from logistic regression. We decided to utilize bootstrapping as it requires less distributional assumptions and utilizes a larger sample size as compared to other methods (split sample or jackknife). The BCa method adjusts for bias in the bootstrapped sampling distributions [27].

We performed nested logistic regression and computed sequential partial R tests to assess the relative contribution of adding the variables included in the final model. The first partial R test was calculated when all identified clinical variables were included. The second partial R test was calculated after adding the radiological findings. The final R test was calculated after we forced two important social risk factors (prior imprisonment and health care workers). We chose this strategy because the radiological variable was available only in a subset of patients and because prior imprisonment and health care workers have been associated with MDR-TB [21], [22], [23]. Finally, we compared AUROCs for these three models.

We used STATA 10·1 software for all statistical analyses (StataCorp, College Station, TX, USA).

## Results

Among 964 patients with proven pulmonary TB, 82 had MDR-TB (prevalence, 8.51%; 95% confidence interval, 6.75%–10.27%). Complete data on all covariates was available for 75·6% (729/964). Table 1 presents the demographic, clinical and radiographic characteristics of the derivation sample.

**Table 1:** Study Population Characteristics.

Variable*	Total (N = 964)	MDR-TB (n = 82)	Non- MDR-TB (n = 882)	p-value
Demographics				
Age, years, mean ± SD	30·6±10·5	29·7±10·9	30·6±10·5	0·44
Gender, male	583 (60·5%)	51 (62·2%)	532 (60·3%)	0·74
Risk factors				
History of tuberculosis†	70 (7·3%)	33 (40·2%)	37 (4·2%)	<0·001
MDR-TB contact, family	89 (9·2%)	15 (18·3%)	74 (8·4%)	0·003
MDR-TB contact, other	117 (12·1%)	16 (19·5%)	101 (11·5%)	0·03
TB, family death	78 (8·1%)	12 (14·8%)	66 (7·5%)	0·02
TB contact	529 (54·9%)	48 (58·5%)	481 (54·5%)	0·49
History of imprisonment	58 (6%)	3 (3·7%)	55 (6·3%)	0·35
Health care worker	42 (4·4%)	4 (4·9%)	38 (4·3%)	0·81
Co-morbidities				
Alcohol use	146 (15·2%)	4 (4·9%)	142 (16·1%)	0·007
Smoker	123 (12·8%)	2 (2·4%)	121 (13·7%)	0·003
Drug use	84 (8·7%)	7 (8·5%)	77 (8·7%)	0·95
Diabetes mellitus	20 (2·1%)	2 (2·4%)	18 (2%)	0·81
HIV/AIDS	3 (0·3%)	0 (0%)	3 (0·3%)	0·60
Symptoms				
Decreased appetite	497 (51·8%)	36 (44·4%)	461 (52·5%)	0·17
Weight loss	714 (74·4%)	51 (63·0%)	663 (75·4%)	0·01
Fever, sweat, or chills	773 (80·2%)	58 (70·7%)	715 (81·1%)	0·03
Cough, productive	890 (92·7%)	71 (87·7%)	819 (93·2%)	0·07
Hemoptysis	418 (43·5%)	37 (45·7%)	381 (43·3%)	0·67
Dyspnea	685 (71·4%)	52 (64·2%)	633 (72%)	0·14
Physical Exam Findings				
Weight, Kg, mean (SD)	55·3±9	56·5±9·7	55·2±9	0·19
Systolic blood pressure, mmHg, mean (SD)	101·3±13·6	104·4±14·3	101±13·5	0·03
Lung, abnormal	773 (80·6%)	60 (74·1%)	713 (81·2%)	0·12
Cardiac, abnormal	118 (12·3%)	4 (4·9%)	114 (13%)	0·04
Abdominal, abnormal	23 (2·4%)	0 (0%)	23 (2·6%)	0·14
Skin, abnormal	483 (50·3%)	34 (42·0%)	449 (51·1%)	0·12
Chest radiograph pattern				
Cavitary	98 (13·4%)	13 (25·5%)	85 (12·5%)	0·009
Alveolar	116 (15·9%)	5 (10·0%)	111 (16·3%)	0·24
Reticular	90 (12·3%)	8 (15·7%)	82 (12·1%)	0·45
Nodular	13 (1·8%)	0 (0%)	13 (1·9%)	0·32

*Data is expressed as n (%) or mean ± SD.

† Failed therapy or early relapse within 12 months.

### Score Derivation

In the bivariate analysis, we found an association at p<0·20 between pulmonary MDR-TB and four risk factors, two co-morbidities, five symptoms, seven physical exam findings, and one radiographic pattern (Table 1).

In the logistic regression analysis, variables retained in the full model included prior TB, MDR-TB contact within family, abnormal lung exam, and cavitary pattern in the chest radiographs (adjusted R2 = 21.%, p<0·001). The AUROC was 0.76 (95% CI 0.68 to 0.84).

Table 2 presents the derived risk score. The prevalence of MDR-TB increased by clinical probability category, low (3.2%), intermediate (6.0%), and high (41.8%) (p<0.001), Table 3. Similarly, the likelihood ratios (LR) increased by clinical probability category, (LR 0.4), intermediate (LR 0.8), and high (LR 9.6), Table 3.

**Table 2:** Multidrug-Resistance Pulmonary Tuberculosis (MDR-TB) Score.

Variable	Regression Coefficients (95% BCa CI)	Points
Risk factors		
History of tuberculosis*	2·78 (1·98 to 3·49)	3
MDR-TB contact, family	1·27 (0·34 to 2·15)	2
Physical exam		
Abnormal lung exam	−0·82 (−1·57 to −0·12)	−1
Chest radiograph pattern		
Cavitary	0·93 (0·14 to 1·66)	1

BCa  =  Bias-Corrected and accelerated, CI: confidence interval.

*Failed therapy or early relapse within 12 months.

**Table 3:** Proportion of Patients Classified by the MDR-TB Score and Likelihood Ratios.

Clinical Probability Category	Patients n (%)	MDR-TB Prevalence (Pretest Probability) n (%)	Likelihood Ratio (95% CI)
Low(0 points)	440 (60·4)	14 (3·2)	0·4 (0·3 to 0·6)
Intermediate(1–2 points)	234 (32·1)	14 (6)	0·8 (0·5 to 1·3)
High(>2 points)	55 (7·5)	23 (41·8)	9·6 (6 to 14·8)
All	729 (100)	51 (7)	

MDR-TB: Multidrug-resistant tuberculosis, CI: confidence interval.

Selecting a cut-off score of one or greater, resulted in a sensitivity of 72.6%, specificity of 62.8%, LR positive of 1.95, and LR negative of 0.44 (correctly classified 63.5%). Similarly, selecting a cut-off score of two or greater, resulted in a sensitivity of 60.8%, specificity of 87.5%, LR positive of 4.85, and LR negative of 0.45 (correctly classified 85.6%). Finally, selecting a cut-off score of three or greater, resulted in a sensitivity of 45.1%, specificity of 95.3%, LR positive of 9.56, and LR negative of 0.58 (correctly classified 91.8%).

A history of prior TB had a sensitivity of 40.2% and a specificity of 95.8%; a history of MDR-TB contact within family had a sensitivity of 18.3% and a specificity of 91.6%.

### Model Validation

We did not observe a significant discrepancy between the observed and predictive number of patients with MDR-TB (p = 0.39), indicating an adequate goodness-of-fit for the full model. The bias-corrected and accelerated (BCa) 95% confidence intervals estimated from bootstrapping are shown in Table 2; the bounds were similar as compared to the 95% confidence intervals from the logistic regression model (data not shown).

The nested logistic regression illustrated the added explanatory power of the covariate blocks. The adjusted R2 for the model with clinical variables (prior TB, MDR-TB contact in family, abnormal lung exam) was 18.8% (p<0·001); the explanatory power increased by the addition of the radiologic cavitary pattern (adjusted R2 = 20.2%, p = 0.02); and finally, the explanatory power did not increase by forcing the two social risk factors, history of imprisonment and health care worker (adjusted R2 = 21.3%, p = 0.15). Among patients with complete data (n = 729), the AUROC for the models with the clinical variables alone (0.75, 95% CI 0.67 to 0.83), the clinical and radiologic pattern (0.76, 95% CI 0.68 to 0.84) and the full model (0.77, 95% CI 0.69 to 0.85) were not statistically different (p = 0.6). Among patients with complete data on the clinical variables (n = 964), the AUROC was 0.72 (95% CI 0.65 to 0.78).

## Discussion

Our findings suggest that a simple CPR can stratify the risk for MDR-TB among patients with pulmonary TB in an endemic area. The rule includes and combines four readily available variables: MDR-TB contact within family, history of prior TB, having an abnormal lung exam, and cavitary patterns on the chest radiographs. The strongest clinical predictors were previous history of TB (failed or relapsed after the standard regimen) and MDR-TB contact within family.

Our findings are consistent with other studies describing independent risk factors for MDR-TB. Risk factors that have been associated with MDR-TB include: prior TB [18], [19], [20], [21], [22], known TB contacts [20], age younger than 45 years [18], [21], HIV positivity [18], [20], health-care workers [20], [24], and previous imprisonment [21], [22], [23]. We did not find association with age as our population was predominantly young. Similar to other countries in Latin America, the prevalence of HIV co-infection was low, thus assessing the association with MDR-TB was not possible [28]. Even though we did not find statistical association with health care workers and history of imprisonment; we forced them into the model, because these variables have been associated with resistant TB [18], [19], [20], [21], [22], [23], [24]. However, they did not contribute to improve the goodness of fit in the final model. These results might be explained by the low number of patients with these risk factors into the sample. We found that MDR-TB patients are more likely to have cavitary lesions in accordance with previous studies [29], [30]; perhaps because such patients often have had active TB for longer periods of time and the greater prevalence of cavitation may just reflect the prolonged time with active TB. We can only speculate for the seemingly protective effect of an abnormal lung exam finding for MDR-TB risk. A protective effect for MDR-TB indicates a higher risk for sensitive TB; since patients with cavitary lesions are more likely to have MDR-TB, patients with sensitive TB may have an interstitial radiographic pattern that can elicit crackles in the lung exam (this hypothesis warrants further confirmation).

Other CPR models have been developed to assist the decision for respiratory isolation of patients with suspected TB [10], [11], [12], or in the diagnosis of smear-negative pulmonary TB [13], as well as to predict the clinical course among patients with known TB [14]. Clinical features identified in these studies were weight loss, fever or night sweats, hemoptysis, age >45 years old, productive cough, and upper-lobe infiltrate on chest radiograph or cavities.

We are not aware of any other CPR developed specifically to stratify the risk of MDR-TB among patients with TB in endemic or non-endemic areas. We should note a previous study performed in Thailand [15]. Boonsarngsuk and colleagues found that chest radiograph features, relapse after previous treatment, and prior incomplete treatment were associated with an increased risk for either isoniazid or rifampin resistance; a cut-off score of greater than two had a sensitivity of 58% and a specificity of 68% [15]. However, the study was retrospective, had a small sample size (290 patients), patients were selected base on physicians' judgment, patients were treated in a referral hospital, and included microbiological results from invasive procedures (bronchoalveolar lavage fluid); the prevalence of MDR-TB was 2.4% (7 cases) and HIV was 16%. In our study, selecting a cut-off score of two or greater, resulted in a sensitivity of 60.8% and specificity of 87.5%. We built a new CPR by validating previously identified risk factors for MDR-TB [18], [19], [20], [21], [22], [23], [24] as well as clinical and radiographic findings in MDR-TB patients [29], [30] for inclusion in the model; adjusting them for multiple independent factors single score. Furthermore, we identified the relative weighs of independent factors and combined them in a single score. If further validated, such a rule could be used for testing and treatment decisions.

The present study has several strengths: first, the sample size was large and representative of a highly prevalent area for MDR-TB, where patients were enrolled from 37 health care centers. Second, the data was collected prospectively in an operational setting. Third, the culture and DST were done in a reference laboratory that follows the standard WHO guidelines [4], furthermore the personnel performing the DST was unaware of the patients' clinical or radiographic findings. Finally, we followed recommended methodology to develop a CPR, adjusted for independent factors and performed internal validation. Thus, our study fulfills validity criteria for the development of a CPR.

Our study has some limitations. First, while all patients had chest radiographs, not all films were available for interpretations as it was not requirement for the original clinical trial. However, we do not expect selection bias because the AUROC excluding chest radiographs was 0.72 (n = 964) and 0.74 among patients with complete data (n = 729). Second, our findings may not apply to areas with higher prevalence of HIV/TB co-infection; in our setting, all patients with TB are tested for HIV. Finally, similar to other real-world operational settings, we only included patients with positive AFB smear. As compared to culture media, the sensitivity of AFB smear is <80%; however, routine culture in all patients is neither universally accepted nor a feasible practice in developing countries.

In summary, diagnosing MDR-TB requires the use of techniques that are of limited availability and high expense for developing countries, such as culture in liquid media with DST, or line probe assays [31], [32], [33], [34], [35], [36]. Developing improved and affordable diagnostic tools for MDR-TB represent priority areas in public health research. We have developed a CPR based on easily obtainable clinical findings (prior TB, MDR-TB contact, abnormal lung exam) and one radiological pattern (cavity) in patients with pulmonary TB to stratify the risk of MDR-TB in resource-limited and endemic area such as Perú. If further validated, such a tool may help TB programs examine the costs and benefits implications of sensitivity testing, especially in resource-limited endemic areas. For example, in our sample, in the 60.4% of patients who were deemed at low risk according to the rule, the prevalence of MDR-TB was 3.2%. In the Peruvian Tuberculosis Program such patients do not receive culture and sensitivity testing initially, this 3.2% of patients may continue transmission of MDR-TB. Exploring the costs and benefits implications of testing all patients at low risk is warranted. We are not advocating the immediate use and implementation of such a clinical-prediction rule. Finally, cost-effectiveness studies are needed to evaluate the potential impact of this tool in decisions regarding treatment regimens or infection control measures.
